# Studying removal of anionic dye by prepared highly adsorbent surface hydrogel nanocomposite as an applicable for aqueous solution

**DOI:** 10.1038/s41598-024-59545-y

**Published:** 2024-04-20

**Authors:** Aseel M. Aljeboree, Ayad F. Alkaim

**Affiliations:** Department of Chemistry, College of Sciences for Girls, University of Babylon, Hilla, 5001 Iraq

**Keywords:** Hydrogel, Biopolymer, Bentonite clay, Thermodynamics, Kinetics model, Recyclability, Environmental chemistry, Materials chemistry, Polymer chemistry, Chemistry, Materials science, Nanoscience and technology

## Abstract

In this study, a Sodium alginate-g-poly (acrylamide-clay)/TiO_2_ hydrogel nanocomposite [SA-g-p(AM-Bn)/TiO_2_] was synthesized using the biopolymer sodium alginate (SA), acrylamide (AM), and bentonite clay (Bn) as hybrid materials embedded with titanium dioxide nanoparticles (TiO_2_NPs) for the removal of toxic Congo Red (CR) dye from an aqueous solution. The [SA-g-p(AM-Bn)/TiO_2_] nanocomposite has been described on the basis of thermal stability, morphological analysis, estimation of functional group, and crystalline/amorphous character by TGA, EFSEM/EDX, TEM, FT-IR, and XRD analysis, respectively. The effects of operational parameters toward the CR dye adsorption on [SA-g-p(AM-Bn)/TiO_2_], including contact time, adsorbent dosage, initial concentration, initial pH, and temperature were investigated. The maximum adsorption efficiency was found to be 185.12 mg/g for [SA-g-p(AM-Bn)/TiO_2_] in 100 mg/L of solution CR at pH 6.0 within 1 h. The equilibrium isotherms, kinetics, and thermodynamics parameters of adsorption were examined, and results showed that the isotherm fitted the Freundlich model and the kinetics adsorption model of CR followed pseudo-first-order, thus indicating physisorption of anionic-CR onto the sorbent due to the development of an electrostatic attraction bond. Thermodynamic parameters for [SA-g-p(AM-Bn)/TiO_2_] have values (ΔG and ΔH) reflecting the spontaneous and endothermic nature of the adsorption processes. Moreover, [SA-g-p(AM-Bn)/TiO_2_] presented outstanding excellent reusability and recyclability with a relatively best removal percentage as compared to [SA-g-p(AM-Bn)] and suggested their applicability towards the textile industry and water purification purposes.

## Introduction

Dyes are highly toxic compounds that can cause permanent damage to the skin and eyes. Textile dyes are widely used in several industries to color final products. Dyes contain aromatic rings in their structures, consisting of either auxochromes or chromophores. Auxochromes (e.g. N=N, NO, NO_2_) enhance the action of chromophores, which are responsible for color production (e.g. NH_2_, OH, COOH, NR_2_, NHR, Cl). Together, these groups improve solubility in water and binding affinity. Conventional methods for removing dyes from wastewater include membrane filtration, adsorption, photochemical degradation, electrochemical destruction, ion exchange, and anaerobic bioremediation^1–4^.

Congo Red (CR) dye, disodium 4-amino-3-[4-[4-[4-(1-amino-4-sulfonatonaphthalen-2-yl)diazenyl]phenyl]diazenyl]naphthalene-1-sulfonate, is an aromatic diazo dye containing two azo groups (–N=N–) that enable coloration. It exhibits high stability, toxicity, and resistance to biodegradation. CR dye is produced by textile, paper, printing, and other industries. As an anionic benzidine derivative, Congo Red is toxic to many organisms and a known carcinogen owing to its complex aromatic structure. Adsorption techniques can effectively remove CR dye from contaminated wastewater^5–12^.

Hydrogels have attracted significant research attention owing to their advantageous properties, including high water retention, hydrophilicity, cost-effectiveness, biocompatibility, and responsiveness to environmental conditions (e.g. temperature, ionic strength, pH). Hydrogels consist of cross-linked 3D polymer networks. Alginate is a naturally derived polysaccharide commonly utilized in drug delivery, cell encapsulation, and injectable cell transplantation due to its favorable properties^13–16^.

Sodium alginate (SA) consists of poly-β-1,4-D-mannuronic acid and α-1,4-L-guluronic acid residues. Its structure contains hydroxyl and carboxyl groups, which can undergo crosslinking with agents like ions to form hydrogels. Alginate can be extracted from its salt forms via ion exchange. In di- and monovalent cations, water-soluble alginates transform into water-insoluble salts. The -OH and -COO- groups on the alginate backbone enable the adsorption of dye pollutants. However, like other polysaccharides, sodium alginate suffers from poor stability. To improve stability and adsorption efficiency, alginate can be cross-linked with synthetic polymers to form configurationally stable hydrogels for applications as adsorbents without dissolution in water^17–20^.

Titanium dioxide (TiO_2_) is widely used in applications such as photocatalysis, energy storage and conversion, cosmetics, etc., owing to properties like abundance, inertness, chemical/physical stability, water insolubility, low toxicity, and the ability to photodegrade various dyes^13,21^. However, TiO_2_ has limitations, including a low adsorption capacity and a high aggregation tendency. Incorporating additional adsorbents can augment the dye removal performance. Biopolymers and clays are suitable candidates in this regard. Clays like kaolinite, hallosite, and bentonite have been employed as TiO_2_ supports to synthesize TiO_2_-clay nanocomposites. Recovering TiO_2_ and clay nanoparticles in powder form is challenging after water treatment^22^. Encapsulating the nanocomposite within a biopolymer matrix enables easier recovery while enhancing removal capacity. In summary, the synergistic effects of TiO_2_, clay minerals, and biopolymers can potentially be leveraged to design efficient and recoverable adsorbent nanocomposite systems for water remediation^23–27^.

Although the hydrogels derived from synthetic polymers are bestowed with good adsorptive, swelling, and mechanical properties, the underlying polymers are costly, non-biodegradable, and toxic. In contrast, biopolymer-derived hydrogels are less expensive, non-toxic, and biodegradable, but their poor mechanical strength hinders their large-scale applications. Therefore, the incorporation of biopolymers into the synthetic hydrogels may combine the beneficial features of both precursors^28–30^. Also, hydrogel composites are developed by the incorporation of numerous clays into the hydrogels. In this domain, hybrids containing alginates and bentonite clays are extensively researched owing to their promising adsorption profiles, low cost, natural abundance, and environmentally benign nature. For instance, Sodium alginate-g-poly(acrylic acid-co-2-hydroxyethyl methacrylate)/montmorillonite^31^, Sodium alginate grafted poly(N-vinyl formamide-co-acrylic acid)-bentonite clay^32^ and sodium alginate grafted poly (acrylic acid-co–N-vinyl formamide)^33^, Sodium Alginate Graft Poly(Acrylic Acidco-2-Acrylamide-2-Methyl Propane Sulfonic Acid)/ Kaolin^34^, Sodium alginate/bentonite impregnated TiO_2_^25^, sorbents of admirable swelling and sorption properties have been developed. Hence, biopolymer sodium alginate (SA) was incorporated into the synthetic hydrogel to reduce toxicity and enhance swelling and sorption potencies.

In this study, it is intended to fabricate a Sodium alginate -g-poly (acrylamide-bentonite clay)/TiO_2_ nanocomposite, [SA-g-p(AM-co-Bn)/TiO_2_] nanocomposite hydrogel. By using the biopolymers sodium alginate (SA), acrylamide (AM), and bentonite clay (Bn) embedded with TiO_2_NPs. The [SA-g-p(AM-co-Bn)/TiO_2_] nanocomposite hydrogel has been described on the basis of thermal stability, morphological analysis, estimation of functional group, and crystalline/amorphous character by TGA, FESEM/EDX, TEM, FT-IR, and XRD analysis, respectively. To enhance the removal efficiency of CR dye in an aqueous solution, the study examined the effects of various optimization parameters like contact time, adsorbent dose, initial pH, and temperature of the adsorption medium. Adsorption was analyzed through adsorption kinetics and isotherm equilibrium studies. The recyclability of the samples was tested through various adsorption–desorption cycles.

## Materials and methods

Sodium alginate (SA, Formula: C_6_H_9_NaO_7_, Molar mass: 216.12 g/mol, purity: 99.8%), Bentonite clay Purity: 99.9%, Calcium chloride dihydrate (Formula:CaCl_2_·2H_2_O, Molar mass:147.995 g/mol, Purity: 98.9%), Methanol (Formula: CH_3_OH, Molar mass: 32.04 g/mol, Purity:99.9%), Ethanol (Formula: C_2_H_5_OH, Molar mass: 46.07 g/mol, Purity:99.9%), Congo red dye (CR) (Formula: C_32_H_22_N_6_Na_2_O_6_S_2_, Molar mass: 696.66 g/mol, Purity:98.8%), Titanium (IV) bis(ammonium lactate) dihydroxide (TALH) (Formula: [CH_3_CH(O–)CO_2_NH_4_]2Ti(OH)_2_, Purity:99.9%). Hydrochloric acid (HCl) Molar mass: 37.46 g/mol), Sodium hydroxide (NaOH), (Molar mass: 39.997 g/mol, Purity: 99.9%), phosphoric acid (H_3_PO_4_) Molar mass: 97.9 g/mol, Purity:99.9%), Sulfuric acid (H_2_SO_4_), Molar mass 36.07 g/mol, Purity:99.9%), Nitric acid, HNO_3_, Purity:99.9%, were all analytical grade and obtained from Sigma Aldrich.

### Preparation of the TiO_2_ NPs

Titanium dioxide nanoparticles (TiO_2_ NPs) were prepared by thermal hydrolysis of titanium (IV) bis(ammonium lactate) dihydroxide (TALH) in a stainless steel autoclave. 10 mL of TALH solution was mixed with 100 mL of distilled water and 0.1 M ammonium hydroxide (NH_4_OH) in a 250 mL Teflon cup. The resulting mixture was stirred for 10 min to ensure thorough mixing. The Teflon cup was then sealed in the autoclave and placed in an electric furnace at 160 °C for 24 h. After thermal hydrolysis, the autoclave was cooled to room temperature, and the TiO_2_ NP powder was collected by high-speed centrifugation (at least three cycles) and washed with deionized water (at least four times). Finally, the TiO_2_ NPs were dried overnight in an oven at 60 °C^35^.

Preparation of the sodium alginate -grafted-poly (acrylamide-bentonite clay) TiO_2_ hydrogel nanocomposite.

**Figure 1:**
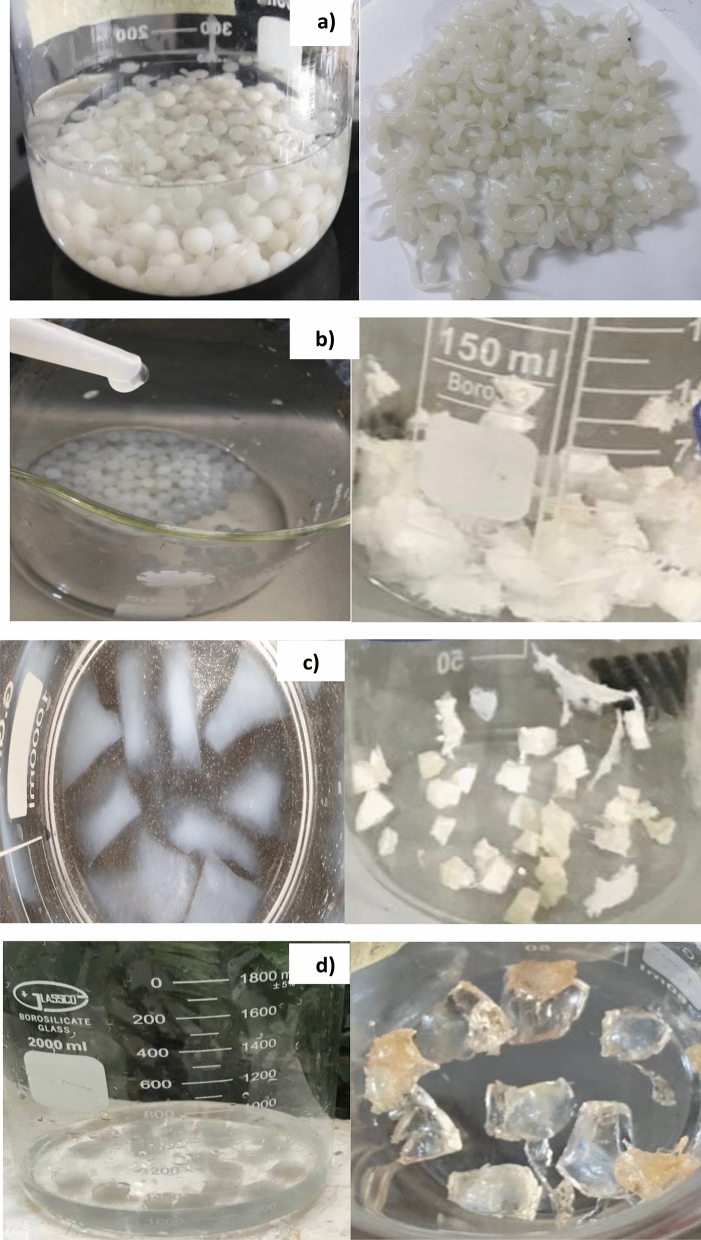
Real image of the sodium alginate -g-poly (acrylamide- Bentonite clay)/TiO_2_ nanocomposite (**a**) SA:AM:Bn (4:2:2), (**b**)SA:AM:Bn (3:3:1.5), (**c**) SA:AM:Bn (2:4:1), (**d**) SA:AM:Bn (1:5:0.5).

A sodium alginate-grafted -grafted-poly (acrylamide-bentonite clay) TiO_2_ hydrogel nanocomposite SA-g-P(AM-BC)/TiO_2_ nanocomposite was synthesized. Different amounts of SA (1–4) g were dissolved in 150 mL of deionized water and added and stirred for 2 h at 45 °C. Variable amounts of acrylamide (AM) monomer (2–5) g were dissolved in 50 mL of deionized water. Then, variable amounts of bentonite clay (0.5–2) g were dissolved in 50 mL of deionized water and stirred for 1 h. The prepared bentonite clay dispersion was then gradually added to the SA and AM solution mixture and further stirred at 25 °C for 2 h to obtain a homogeneous dispersion gel. In the second step, surface functionalization with TiO_2_ nanoparticles, the polymer matrix dispersion prepared in step 1 was added dropwise using a syringe needle to a bath solution composed of 0.5 w/v% TiO_2_ nanoparticles and 4 w/v% CaCl_2_.2H2O This simultaneous ionic crosslinking with Ca^2+^ ions and surface impregnation with TiO_2_ nanoparticles yielded the final biopolymer nanocomposite. Note: The best weight ratios of biopolymer were (4 g) SA, (2 g) AM and Bn clay (2 g), as the resulting beads were able to swelling to a very large compared to the rest of the preparation ratios, as shown in Fig. 1.

### Adsorption studies

Congo red (CR) dye adsorption onto SA-g-p(AM-Bn)/TiO_2_ was performed by UV–vis spectroscopy. Several adsorption parameters, for example, adsorbent dose of SA-g-p(AM-Bn)/TiO_2_, equilibrium time, initial pH, and temperature at the best optimum conditions, Spectrophotometric analysis was carried out through UV–visible spectra with the help of a double-beam spectrophotometer at 495 nm, and deionized water was used as a reference solvent. For a specific adsorption of CR dye, 0.05 g of SA-g-p(AM-Bn)/TiO_2_ was utilized in 100 mL of CR dye solution at 100 mg/L for 1 h. The solution was shaken at 200 rpm in a shaker water bath. Maximum adsorption capacity Qe (mg/g) and removal percentage E% were calculated by using Eqs. (1) and (2):

1 $${\text{Qe}}=\frac{{({\text{C}}}_{0}-{{\text{C}}}_{{\text{e}}})\times {{\text{V}}}_{{\text{L}}}}{{{\text{m}}}_{{\text{g}}}},$$

2 $$\mathrm{E \%}=\frac{{{\text{C}}}_{0-}{{\text{C}}}_{{\text{e}}}}{{{\text{C}}}_{0}}\times 100,$$

where Qe is the amount adsorbed at equilibrium (mg/g), Co and Ce are the initial and equilibrium liquid phase concentrations of CR dye (mg/L), V is the volume of CR dye solution (L), and W is the mass of adsorbent used (g).

### Recyclability study

Adsorption–desorption analysis was utilized to estimate the reusability of SA-g-p(AM-Bn)/TiO_2_. The CR-adsorbed SA-g-p(AM-Bn)/TiO_2_ was recyclable and regenerated via washing at various concentrations (0.01–0.1 N) of NaOH, H_2_SO_4_, HCl, H_3_PO_4_, HNO_3_, acetone, ethanol, and water. The collected SA-g-p(AM-Bn)/TiO_2_ adsorbent was again rinsed via deionized water and dried at 65 °C for 12 h. After that, SA-g-p(AM-Bn)/TiO_2_ was again utilized for the removal of CR dye.

### Absorbency and swelling in water

The synthesized biopolymer nanocomposite's water absorbency and swelling properties were examined at 25 °C. The dry mass of the sample was recorded. The sample was then immersed in deionized water to reach equilibrium swelling. The swollen sample was removed, filtered using a clamp to eliminate excess water on the surface, and weighed. The swelling ratio (%SR) was calculated using Eq. (3):

3 $$\mathrm{\%\, Swelling\, Ratio\, }(\mathrm{\%SR})=\frac{{{\text{M}}}_{1}-{{\text{M}}}_{2}}{{{\text{M}}}_{2}} \times 100,$$

where M_1_ is the dry mass before swelling, and M_2_ is the mass after equilibrium swelling in water. This analysis provided insights into the water absorbency characteristics of the synthesized superabsorbent biopolymer nanocomposite.

## Result and discussion

### Characterization for adsorbent/adsorbate

**Figure 2:**
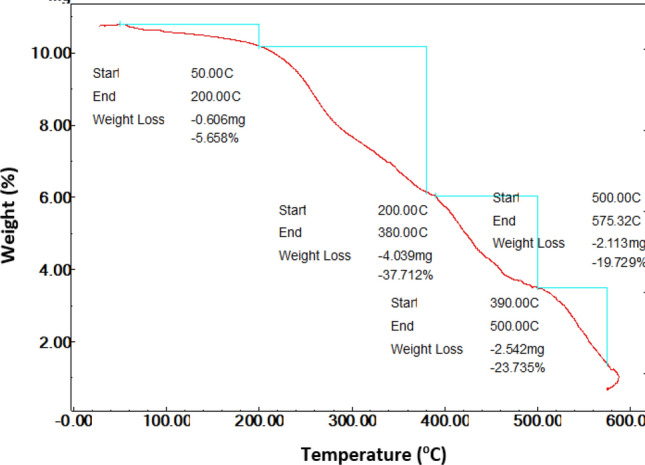
TGA curve of the SA-g-p(AM-Bn)/TiO_2_ nanocomposite.

The thermalgravimetric analysis (TGA) of the SA-g-p(AM-Bn)/TiO_2_ nanocomposite was studied. TGA curves of the nanocomposite obtained at a rate of heating of 50 °C/min up to 600 °C under a dry N2 flow appear (Fig. 2); one can see that the degradation method is different. It is well known that any weight loss below 200 °C is due to the loss of water unbound, while the loss in the range of 200 to 600 °C is mainly due to the degradation of organic matter. By analyzing the TGA of nanocomposite, it is quite clear that the incorporation of clay and TiO_2_ NPs has an approving effect on the thermal stability of nanocomposite SA-supported bentonite^25,36^. This means that clay created a resistant path through the nanocomposite matrix to retard the decomposition process. Similarly, we can detect an improvement in thermal stability attributed to the loading of the TiO_2_ duo via the presence of inorganic and organic materials at their surface. that cause bonds between the Ti and COO groups of the biopolymer. The size reduction and area increase of TiO_2_ NPs on the nanocomposite assure good interactivity among them, thus making complexes more stable^13,25,36,37^.

**Figure 3:**
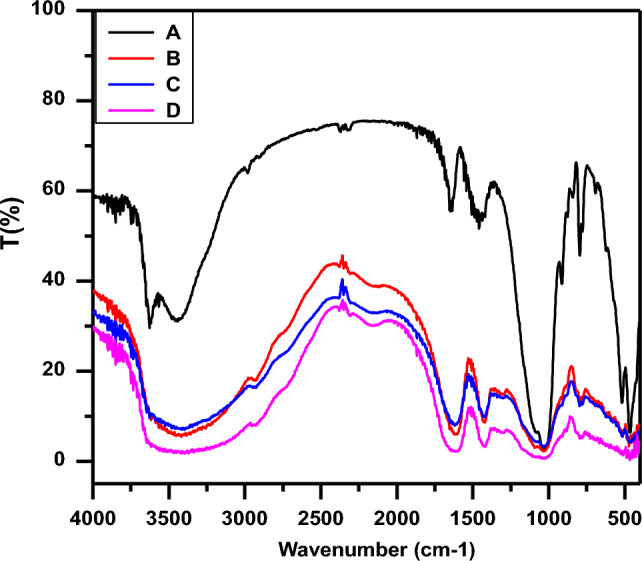
FT-IR spectra of (A) Bentonite Clay, (B) SA-g-p(AM-Bn), (C) SA-g-p(AM-Bn)/TiO_2_ and (D) SA-g-p(AM-Bn)/TiO_2_ surface after adsorption of CR dye.

The Fourier-Transform Infrared Spectroscopy FTIR spectra were shown in Fig. 3, as we see (A) bentonite clay, (B) sodium alginate-grafted polyacrylamide/bentonite clay (SA-g-PAM/BC), (C) SA-g-PAM/BC/TiO_2_ NPs nanocomposite, and (D) SA-g-PAM/BC/TiO_2_ NPs after Congo red (CR) dye adsorption^36,38^. The broad band at 3435 cm^–1^ and 2927 cm^–1^ corresponds to O–H stretching and C–H stretching vibrations. For the nanocomposite (spectrum C), characteristic peaks are observed at 3300 cm^–1^ (O–H stretching), 1600 cm^–1^ (C=O stretching), and 1409 cm^–1^ (symmetric COO– stretching). Bentonite clay (spectrum A) shows typical bands at 1009 cm^–1^ (Si–O–Si stretching), 918 cm^–1^ (Al–OH vibration), and 450 cm^–1^ (Si–O bending). Additional peaks at 1415 cm^–1^ (Ti–O–Ti vibration) and 450–600 cm^–1^ (Ti–O bending) confirm TiO_2_ NP impregnation in the nanocomposite^17,27^.

**Figure 4:**
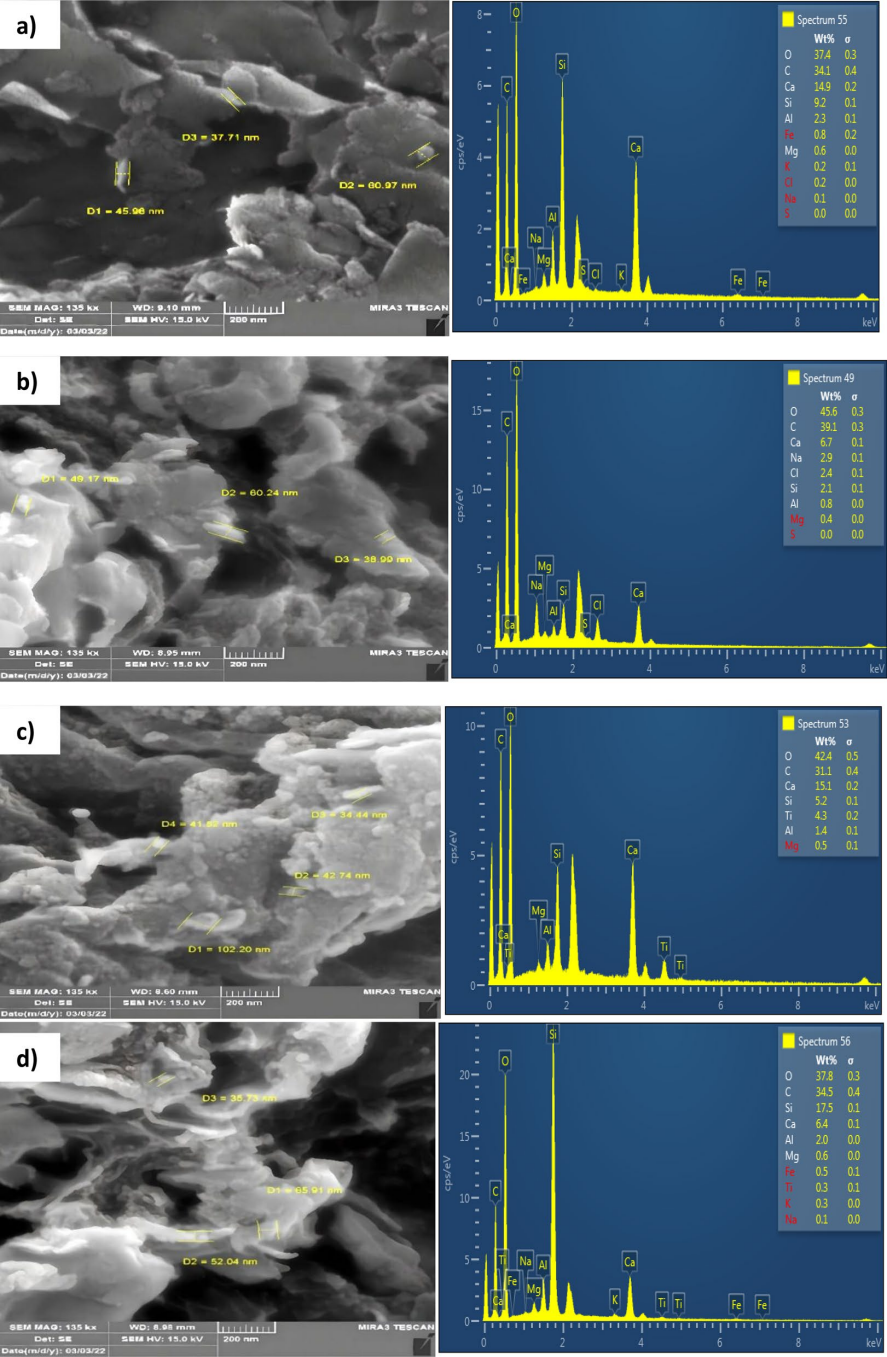
SEM–EDX images of (**a**) Bentonite clay (**b**) SA-g-p(AM-Bn), (**c**) SA-g-p(AM-Bn)/TiO_2_ nanocomposite, (**d**) SA-g-p(AM-Bn)/TiO_2_ nanocomposite after adsorption.

FE-SEM/EDX analysis was conducted to examine the morphology of the surfaces, porous structure, and properties of elemental nanocompositions. Figure 4a–d shows the SEM images along with the EDX analysis. (a) Bn clay; (b) SA-g-p(AM-Bn), (c)SA-g-p(AM-Bn)/TiO_2_ nanocomposite and (d) SA-g-p(AM-Bn)/TiO_2_ nanocomposite before and after adsorption of CR dye. Bentonite clay was found to have a homogeneous and smooth surface with no irregularities (Fig. 4a). It can be seen from Fig. 4b that the surface morphology appeared to be smooth with visible cavities. The SA-g-p(AM-Bn) hydrogel smoothness is a vital parameter that affects the adsorption capacity since it leads to increased hydrophilicity groups. Furthermore, the swelling of hydrogel can be contributed to by the addition of AM. In fact, AM is a molecule that can form 3D networks easily in the hydrogel. Besides, AM has a precise degree of crystallinity, which clarifies the uneven particles on the hydrogel^39,40^.

The globular particles of several sizes embedded on the surface of the nanocomposite suggest that TiO_2_ NPs were successfully combined into the molecular structure of SA. Thus, the morphology of the surface SA-g-p(AM-Bn)/TiO_2_ nanocomposite was a rough, irregular, and heterogonous surface that was well distributed across the nanocomposite due to the loading of TiO_2_ NPs into the nanocomposite polymeric matrix. The EDX analysis confirms. The presence of O, C, Si, Ca, Mg, and Al and Ti elements in the polymeric matrix of SA-g-p(AM-Bn)/TiO_2_. From EDX analysis, the presence of the Ti element with other present elements of nanocomposite reconfirmed the successful incorporation of TiO_2_ NPs in the polymeric matrix of SA-g-p(AM-Bn)/TiO_2_ as shown in Fig. 4c^41^.

In line with the CR dye molecules adsorbed on the SA-g-p(AM-Bn)/TiO_2_ surface, the SA-g-p(AM-Bn)/TiO_2_ surface after adsorption of CR (Fig. 4d) appeared to be more compact with fewer cavities. In addition, increasing elements C and O in the EDX analysis, which belong to CR, reaffirmed the CR dye's molecules being adsorbed on the surface nanocomposite^42,43^.

**Figure 5:**
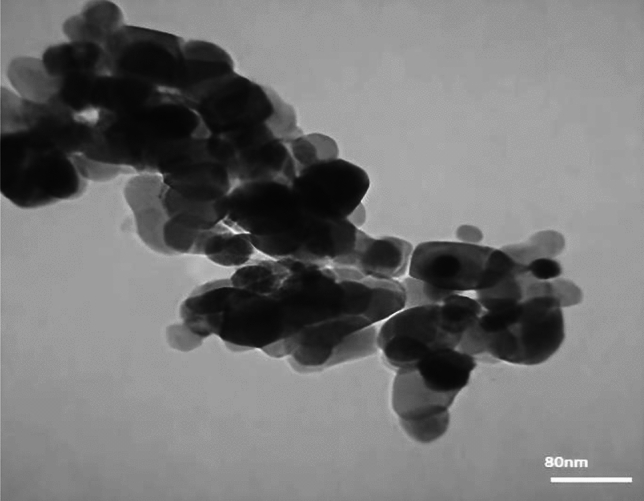
TEM image of SA-g-p(AM-Bn)/TiO_2_ nanocomposite.

The transmission electron microscopy (TEM) image shown in Fig. 5. This figure shows the TiO_2_ NPs embedded within the hydrogel matrix. It can be seen that SA-g-p(AM-Bn)/TiO_2_ appears as regular balls along with some patchy black shapes and tends to form chain-like totals at 80 nm. Moreover, the surface of the SA-g-p(AM-Bn)/TiO_2_ is covered via a transparent layer, where TiO_2_ NPs were observed embedded inside the SA-g-p(AM-Bn)/TiO_2_ and TiO_2_ NPs play a pivotal role in improving stability and increasing surface area as a requisite constituent of synthesizing eco-friendly nanocomposite^2,25,37^.

**Figure 6:**
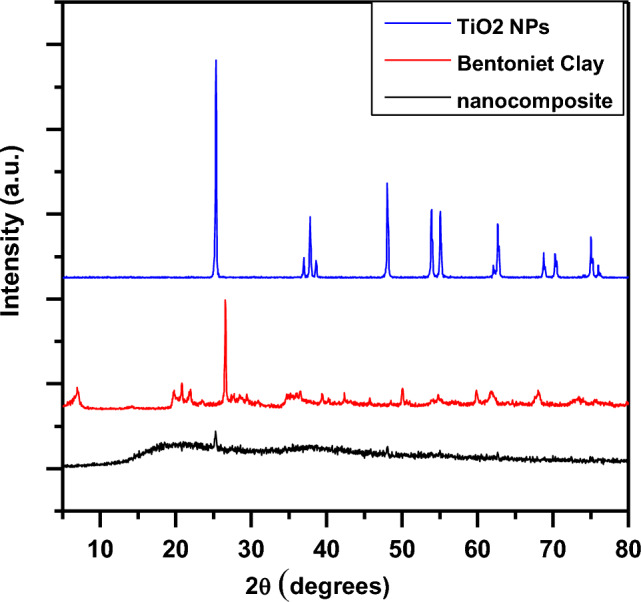
X-ray diffraction patterns for TiO_2_ NPs, Clay Bentonite, SA-g-p(AM-Bn)/TiO_2_.

The XRD patterns in Fig. 6 display characteristic peaks corresponding to (a) TiO_2_ NPs, (b) bentonite clay, and (c) the SA-g-p(AM-Bn)/TiO_2_. Minor variations in clay peak intensities may arise from interactions at the TiO_2_-clay interface. Additionally, strong diffraction peaks at 2θ = 25°, 37°, 48°, 54°, 55°, and 63° match standard TiO_2_ NP signals, confirming the presence of nanocrystalline TiO_2_ phases^25,44^. The observed peaks at 25°, 37°, 48°, 37.97°, 48°, and 54° in the nanocomposite verify the successful integration of TiO_2_ NPs within the biopolymer matrix. While the intensities are somewhat reduced and peak widths slightly broader compared to pristine TiO_2_ due to the amorphous hydrogel coating, the retention of crystalline TiO_2_ signatures demonstrates that the TiO_2_ nanostructures are intact but interacting closely with the polymer network. Thus, XRD analysis verifies the composite nature of the synthesized nanomaterial, which contains crystalline TiO_2_ nanoparticles interspersed in the hydrogel matrix^13,27,42,45^.

### Effect of different parameter

#### Effect of pH

**Figure 7:**
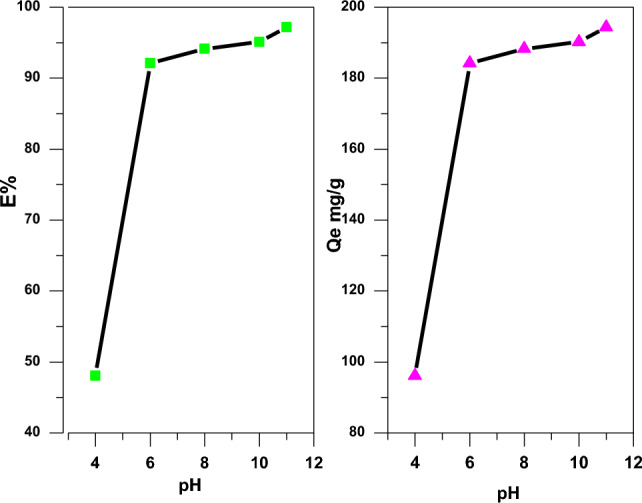
Effect of solution pH on the adsorption of CR dye on SA-g-p(AM-Bn)/TiO_2_. (Exp. Condition: contact time 1 h, T 30 °C, and pH 6.6).

The pH value of the solution affects the adsorption process, depending on the nature of the dyes as well as that of the nanocomposite particles. SA-g-p(AM-Bn)/TiO_2_ particles exhibit high hydrophilicity due to the incorporation of monomers and clay. The hydrogel groups and carboxylic acid groups of nanocomposite particles are protonated/deprotonated with the pH change of the solution and induce hydrophilicity / hydrophobicity in the hydrogel nanocomposite network. The relations between the removal percentage E% of CR and solution pH are shown in Fig. 7. The removal percentage of CR was increased with an increase in solution pH up to 7^46,47^. After that specific pH value, the removal percentage of CR was increased up to 11. This trend was observed due to nature's anionic CR as well as the presence of hydrogel groups or carboxyl acid groups in nanocomposite particles. In an acidic medium, all hydrogel groups or carboxylic acid groups of nanocomposite were totally deprotonated, and negative charges were induced in the network of hydrogels due to the deprotonation of these groups. The presence of a negative charge inside the nanocomposite disables the uptake of anionic CR dye from an aqueous solution due to its low electrostatic attraction. Thus, a decrease in the removal percentage of CR was observed with a decrease in the solution pH at 4. At low pH, water was expelled out and the sieve size of the nanocomposite was decreased, which also resulted in a decreased uptake of CR molecules. Also at low pH, negatively charged carboxylate groups of nanocomposite particles and anionic CR dye molecules repel each other due to the same charge due to a decrease in the removal percentage of CR. Thus, the best optimum value of pH for the best removal percentage (CR) is at pH 7^48,49^.

### Effect of adsorbent dosage

**Figure 8:**
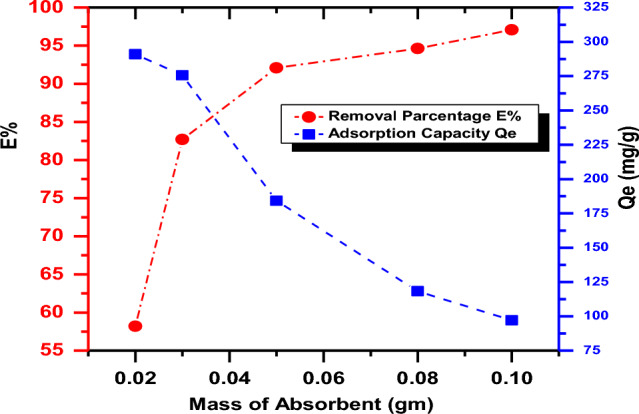
Effect of the mass amount of adsorbent SA-g-p(AM-Bn)/TiO_2_ on the percent removal and amount of adsorbed CR dye, T 30 °C, contact time 1 h., pH = 6.6, initial concentration 100 mg/L.

The influence of the adsorbent dose of SA-g-p(AM-Bn)/TiO_2_ nanocomposite on CR adsorption was examined in the range of different doses (0.02–10.1 g) at 30 °C, pH 7, and 100 mg/L concentration of CR dye (Fig. 8). Increased weight of SA-g-p(AM-Bn)/TiO_2_ results in increased percentage removal of CR dye; the percentage removal was increased from 57.5 to 98.9% for 0.02 g and 0.1 g, respectively. Due to 0.05 g/L of SA-g-p(AM-Bn)/TiO_2_ weight resulted in 92.12% CR dye absorption. The enhancement in removal efficiency with increasing adsorbent weight could be explained by the presence of additional adsorption sites on the adsorbent. Because of the strong competition between the adsorbent and active sites on the adsorbent. An increase in the percentage of dye removal with adsorbent weight was related to increases in the adsorbent surface areas, improving the number of active sites obtainable for adsorption, as reported already in other cases^50,51^. The increase in removal capacity of CR with nanocomposite is due to the introduction of more binding sites for adsorption. The initial parameter explaining this characteristic is that adsorption sites remain unsaturated through the adsorption process, whereas the number of active sites obtainable for adsorption sites increases via increasing the weight of nanocomposite^34,47,51^.

### Effect of temperatures and thermodynamic parameters

To determine whether the ongoing adsorption method was exothermic or endothermic. The adsorption thermodynamics were determined for dye-adsorbent systems. The thermodynamic parameters, including changes in Gibbs free energy (ΔG), enthalpy (ΔH), and entropy (ΔS), were calculated to evaluate the spontaneity and heat aspects of Congo redCr dye adsorption onto SA-g-p(AM-Bn)/TiO_2_ nanocomposite^12,43^.

**Figure 9:**
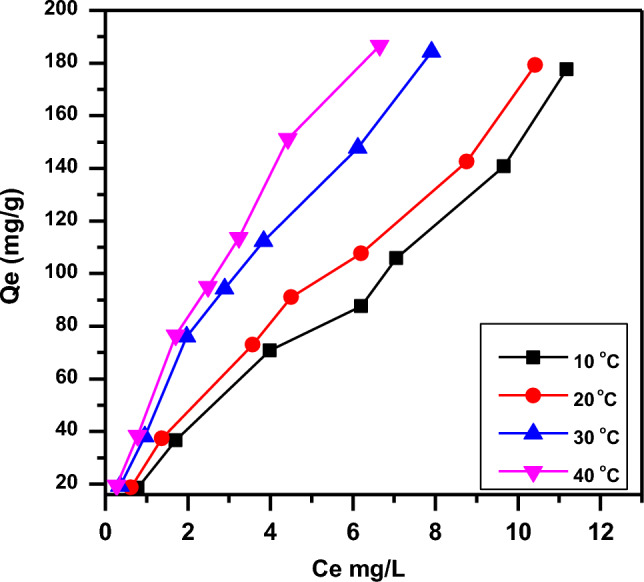
Effect of temperature on the adsorption of CR dye onto SA-g-p(AM-Bn)/TiO_2_ (pH 6.6, mass adsorbent 0.05 g/100 ml).

Batch experiments were performed at varying temperatures (10–40 °C) and concentrations of CR dye (10–100 mg/L) to analyze the effect on adsorption capacity, as shown in Fig. 9. The results indicate that the adsorption capacity (Qe mg/g) of CR dye increased while increasing the solution temperature for all CR dye concentrations. The adsorbent's efficiency of adsorption changes with temperature. Thus, the temperature parameter is important as a physicochemical process. An adsorption endothermic process involves a directly proportional adsorption increase with temperature, caused by an increase in adsorption active sites and the dye molecule's mobility with increasing temperatures. It was found that the increasing temperature of the solution causes a decrease in aqueous phase viscous force resistance, thereby leading to the dye molecule’s faster diffusion across the adsorbent particles' external boundary as well as internal pores. The removal process was also significantly affected by the change in adsorbate molecules' solubility in some cases. At high temperatures, pore size enlargement also causes increased adsorption^1,43,52–54^. Determined of the thermodynamic parameters including (∆H), (∆G), and (∆S) of the adsorption process. The equilibrium constant (K_e_) of the adsorption at each temperature, was calculate via Eqs. (4):

4 $${{\text{K}}}_{{\text{e}}}=\frac{\left({{\text{Q}}}_{{\text{max}}}\right)\times \mathrm{Wt }(0.05\mathrm{ gm})}{\left({{\text{C}}}_{{\text{e}}}\right)\times {\text{V}}(0.1{\text{L}})}\times 1000.$$

The free change energy of adsorption were calculated assuming an activity coefficient of unity for low solute concentrations (Henry’s law) by Eq. (5):

5 $$\Delta {\text{G }} = \, - {\text{RT ln K}}_{{\text{e}}} ,$$

where $\Delta {\text{G}}$: Gibbs free energy (J.K^-1^.mol.^-1^), R is the gas constant (8.314 J.K^–1^.mole^–1^), T is the absolute temperature in Kelvin.

The enthalpy change of adsorption may be obtained from the following Eq. (6):

6 $${{\text{lnX}}}_{{\text{m}}}=-\frac{{\Delta {\text{H}}}^{^\circ }}{{\text{RT}}}+{\text{Cons}}.$$

Calculated, the values of all of the thermodynamic parameters at different temperatures appear in Table 1. The positive values of ΔH and ΔS indicate an endothermic process with increased randomness at the adsorbent-adsorbate interface, whereas the negative ΔG values predict the spontaneous nature of the CR dye adsorption onto SA-g-p(AM-Bn)/TiO_2_ . The decrease in Gibbs free energy with increasing solution temperature also indicates that adsorption is favorable at high temperatures. As temperature rises, the increasingly negative ΔG values predict greater thermodynamic favorability of adsorption at higher temperatures^14,55,56^.

**Table 1:** Thermodynamic parameter ∆H, ∆G, ∆S and, of CR adsorbed on the SA-g-p(AM-Bn)/TiO_2_.

Adsorbent/CR adsorbate				
T/K	Keq	∆G/kJ.mol^–1^	∆H/kJ.mol^–1^	∆S/J.K^–1^.mol^–1^
283.15	14,615.38	− 22.563	17.298	98.786
293.15	17,230.77	− 23.761		
303.15	24,461.54	− 25.455		
313.15	28,461.54	− 26.689		

### Recyclability and reuse study

Evaluating the regenerability and reusability of adsorbents is crucial for viable industrial applications from both economic and environmental standpoints. Desorption studies help elucidate dye removal mechanisms and optimal regeneration strategies for recycling spent adsorbents, lowering operating costs, and mitigating secondary waste pollution. Desorption experiments were performed on dye-loaded nanocomposite using various concentrations (0.01–0.1 N) of NaOH, H_2_SO_4_, HCl, H_3_PO4, HNO_3_, acetone, ethanol, and water as eluents. Remarkably, 100% regeneration was achieved just by using water (Table 2)^57,58^.

**Table 2:** Comparative of desorption removal efficiency of different kind solution for the CR dye on to SA-g-p(AM-Bn)/TiO_2_.

Regeneration and (0.01N)	E%	Regeneration (0.05 N)	E%	Regeneration (0.1 N)	E%
Water	92.09	Water	92.09	Water	92.09
Ethanol	80.12	Ethanol	88.87	Ethanol	90.11
H_3_PO_4_	82.23	H_3_PO_4_	80.22	H_3_PO_4_	77.98
HCl	78.77	HCl	70.77	HCl	66.65
H_2_SO_4_	75.66	H_2_SO_4_	68.11	H_2_SO_4_	55.54
HNO_3_	66.9	HNO_3_	59.65	HNO_3_	40.03
Methanol	60.11	Methanol	57,56	Methanol	39.11
NaOH	52.11	NaOH	42.2	NaOH	36.65

**Figure 10:**
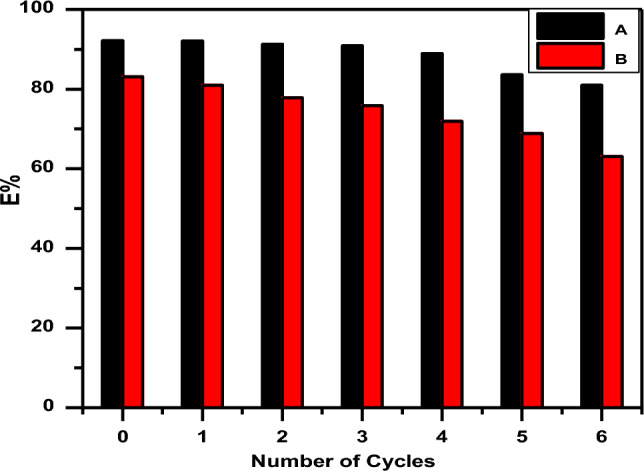
Multi-cycle use of (A) SA-g-p(AM-Bn)/TiO_2_ nanocomposite and (B) SA-g-p(AM-Bn) hydrogel for CR dye adsorption using water as desorption medium.

The SA-g-p(AM-Bn)/TiO_2_ nanocomposite and SA-g-p(AM-Bn) hydrogel matrix were tested for CR dye removal over six repeated adsorption–desorption cycles under optimal conditions (Fig. 10). The adsorption efficiency of the SA-g-p(AM-Bn)/TiO_2_ nanocomposite remains constant through six cycles. In contrast, after four cycles, the SA-g-p(AM-Bn) hydrogel showed a slightly declining performance^57,58^.

The choice of this procedure is associated with the fact that a new equilibrium between the adsorbent and the dye will be established following the formation of new species in water (natural medium pH = 7). The role of water is to increase the CR dye solubility for their interactions on the adsorbent nanocomposite surface. Also, water has a hydrophilic (OH) functional group. This group can firmly adsorb onto the adsorbent nanocomposite surface and interact with the functional group of the nanocomposite^59^. This helps the water molecules firmly adsorb on the adsorbent nanocomposite. In addition, it is also possible that the addition of water may have decreased the interaction extent between the adsorbent nanocomposite surface and CR dye molecules; as a result, CR dye molecules get desorbed from the nanocomposite surface. During the first cycle of adsorption–desorption, regeneration of nanocomposite and hydrogel was effective in desorbing CR (92.81–85.985) and (82.61–68.98%), respectively, after 6 cycles. A possible decrease in percent adsorption after every cycle might be due to the blockage of some active sites by CR dye that are difficult to desorb because of their strong chemical interactions with the nanocomposite surface^60,61^.

### Comparative adsorption study

**Figure 11:**
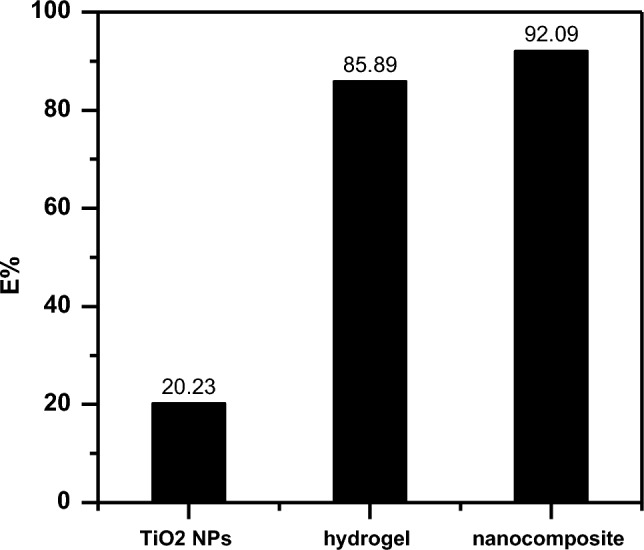
Adsorption of comparative between several surfaces for removal percentage on CR dye.

The CR dye removal efficiencies of TiO_2_ NPs, SA-g-p(AM-Bn) hydrogel, and the SA-g-p(AM-Bn)/TiO_2_ nanocomposite adsorbents were compared via batch experiments. 0.05 g doses of each adsorbent were added to 100 mL solutions of 100 mg/L CR dye concentration and agitated for 1 h. The remaining dye concentrations in the separated supernatants were analyzed by UV–vis spectrophotometry at the maximum absorbance wavelength. As shown in Fig. 11, the nanocomposite exhibits the highest CR removal percentage, outperforming the individual TiO_2_ nanoparticles and hydrogel matrix^25^.

### Adsorption kinetics

The adsorption kinetics were analyzed using pseudo-first order, pseudo-second order, and Elovich models, described by nonlinear Eqs. (7–9)^62,63^.

7 $${\text{qt}} = {\text{qe}}\left[ {{1} - {\text{exp}}\left( {{\text{ k}}_{{\text{f}}} {\text{t}}} \right)} \right],$$

8 $${\text{qt}}= \frac{{\text{K}}2{\text{qe}}2{\text{t}}}{1+{\text{K}}2{\text{qet}}}$$

9 $${\text{qt}}=\frac{1}{\upbeta }1\mathrm{\beta ln}\left(\mathrm{\alpha \beta }\right)+ \frac{1}{\upbeta }\mathrm{lnt },$$

where qt and q_e_ are the adsorbate uptake at time t (mg/g) and at equilibrium (mg/g), k_f_ and k_2_ are the rate constants for pseudo-first order (min^−1^) and pseudo-second order (g/(mg∙min)) models, α is the initial adsorption rate (mg/(g∙min)) and β is the desorption constant (g/mg) in the Elovich model.

**Figure 12:**
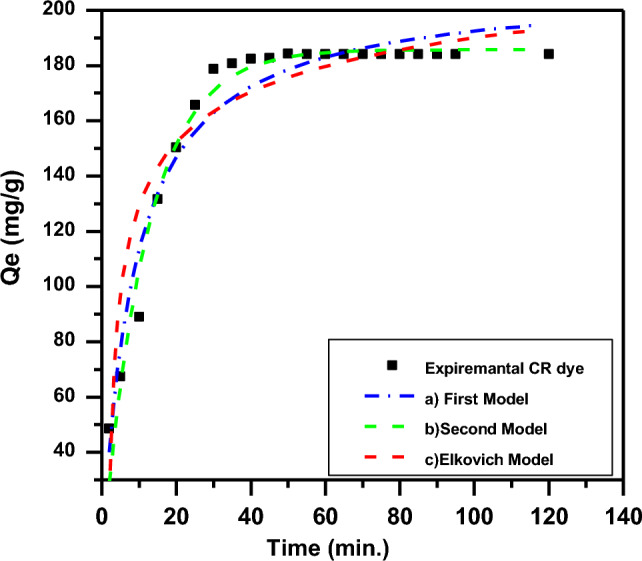
Adsorption rate curve models fitted to experimental CR adsorption onto biopolymer (a) kinetic first model; (b) kinetic second model and (c) kinetic Elkovich model (mass dosage 0.05 g/100 ml, pH 6.6, and T 30 °C).

The kinetic model fits are displayed in Fig. 12, and the parameters are listed in Table 3. The higher correlation coefficients (R^2^ > 0.99) indicate excellent agreement of experimental data with the pseudo-First order model, suggesting chemisorption governs the rate-limiting step for CR dye uptake. Pseudo-second order and Elovich models showed poorer fits^1,11,14,55^.

**Table 3:** First model, second model, and Elovich model including correlation coefficients for CR adsorption on to biopolymer.

Kind	Parameters	Value	Stand. error	*R*2
Pseudo-first-order	q*e* (mg g^−1^)	185.783	1.90388	0.9757
	k*f* (min^−1^)	0.0844	0.0045	
pseudo-second-order	q*e* (mg g^−1^)	208.67	4.8023	0.9466
	ks (gmg^−1^ min^−1^)	0.1188	0.0148	
Elovich	α (mg g^−1^ min^−1^)	88.011	7.6203	0.8717
	β (g min^−1^)	− 1.8810	0.3040	

### Adsorption isotherms

The Langmuir and Freundlich isotherm models were applied to analyze the equilibrium data further. These popular nonlinear isotherm models are described by Eqs. (10) and (11):

10 $${{\text{q}}}_{{\text{e}}}={{\text{K}}}_{{\text{f}}}{{\text{C}}}_{{\text{e}}}^{1/{\text{n}}} ,$$

11 $${{\text{q}}}_{{\text{e}}}=\frac{{{\text{q}}}_{{\text{m}}}{{\text{K}}}_{{\text{L}}}{{\text{C}}}_{{\text{e}}}}{1+{{\text{K}}}_{{\text{L}}}{{\text{C}}}_{{\text{e}}}} ,$$

where qe is the amount adsorbed at equilibrium (mg/g), Ce is the equilibrium concentration (mg/L), and qm and K_L_ are the Langmuir maximum adsorption capacity (mg/g) and affinity constant (L/mg), respectively. K_F_ (mg1^−1^/nL1/ng^−1^) and 1/n are the Freundlich constant and heterogeneity factor.

**Figure 13:**
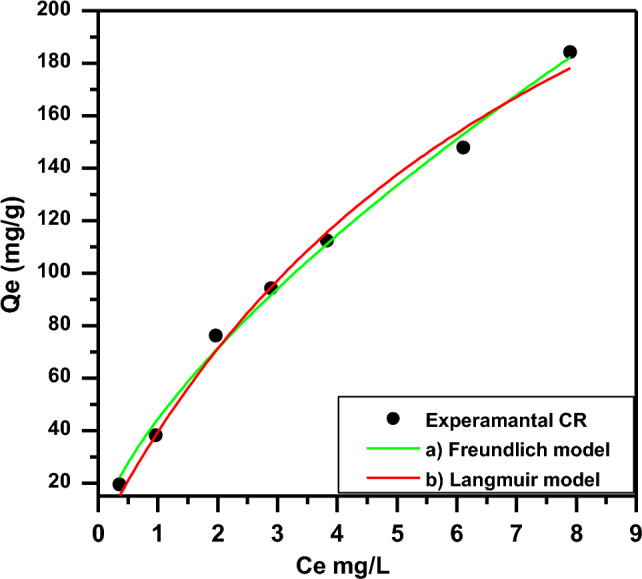
Several adsorptions (a) Freundlich, (b) Langmuir isotherm models nonlinear fit of adsorption CR dye onto biopolymer at 25 °C, pH 6.6, mass of surface 0.05 g/100 mL, and conc. = 100 mg/L).

Figure 13 displays the nonlinear isotherm fits, and Table 4 compiles the model parameters. The excellent correlation coefficient (R^2^ > 0.999) suggests the Freundlich isotherm better represents CR dye binding onto the nanocomposite surface. The 1/n value of 0.683 indicates favorable physicochemical adsorption. The high K_F_ value (44.368) further supports substantial adsorption capacity. While the Langmuir model showed poorer agreement, the maximum monolayer coverage from this approach was calculated as 180.2 mg/g^1,9,64–66^.

**Table 4:** Several factor isotherms for the adsorption study of CR on to SA-g-p(AM-Bn)/TiO_2_.

SA-g-p(AM-Bn)/TiO_2_			
Freundlich ${Q}_{e}={K}_{f}{C}_{e}^{1/n}$	K_f_	44.368	2.241 ±
	1/n	0.683	0.0292 ±
	R^2^	0.9998	
Langmuir ${Q}_{e}=\frac{{q}_{m}{K}_{L}{C}_{e}}{1+{K}_{L}{C}_{e}}$	q_m_ (mg/g)	262.972	40.432 ±
	K_L_ (L/mg)	0.1219	0.0221 ±
	R^2^	0.9911	

### Suggested adsorption mechanism

**Figure 14:**
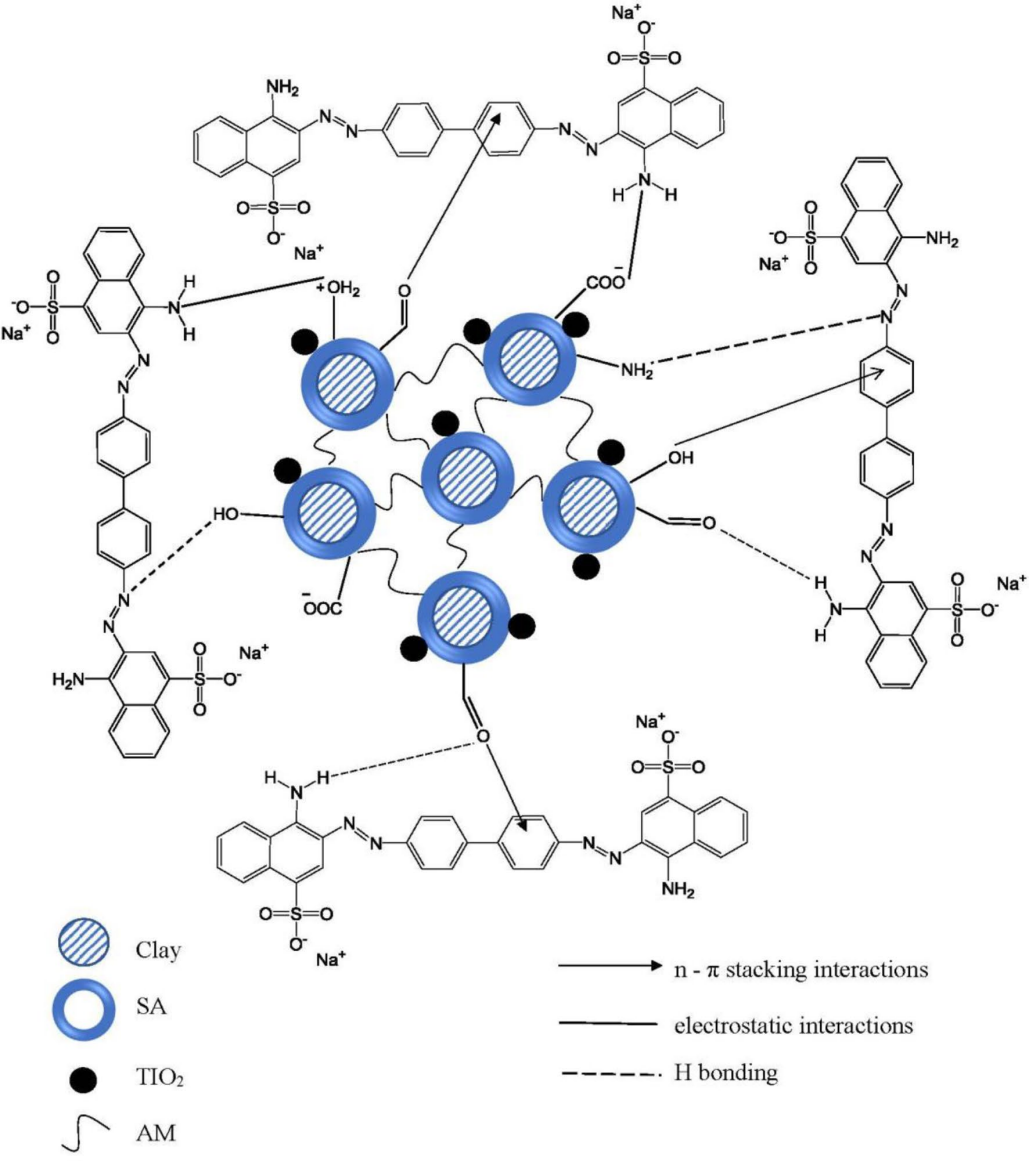
The proposed adsorption mechanism of CR dye via SA-g-p(AM-Bn)/TiO_2_ nanocomposite hydrogel.

The proposed adsorption mechanism of CR dye via SA-g-p(AM-Bn)/TiO_2_ nanocomposite hydrogel has several kinds of interactions, as shown in Fig. 14. There are several functional groups available on nanocomposite surfaces that can adsorb CR dye. These functional groups come from the natural bentonite clay, sodium alginate biopolymer, and titanium dioxide. Thus, the active groups are (–OH), (C=O), (OH_2_^+^), (–NH_2_), (≡Si–OH), (≡Al–OH) and (TiOH_2_^+^), on the surface of nanocomposite. This mechanism involves the electrostatic attraction among negatively charged of (SO_3_^-^) sulfonate groups of CR dye and hydroxyl groups (–OH) and amino groups (–NH_2_)^30^.

Adsorption mechanisms can also contain two kinds of H-bonding, for example, dipole–dipole hydrogen bonding and Yoshida H-bonding. The H-bonding dipole–dipole interactions among free hydrogen of nanocomposite with oxygen and nitrogen in the structure of CR dye, while Yoshida H-bending occurs among the aromatic ring of CR dye and the OH on the nanocomposite surface. Finally, n-π interaction occurs among electron-donating groups of nitrogen and oxygen on the nanocomposite surface and π- system in the aromatic ring of CR dye^67^.

## Conclusion

The synthesized SA-g-p(AM-Bn)/TiO_2_ nanocomposite hydrogel was confirmed via the results of XRD, TGA and EDX. The FESEM micrographs depicted the highly rough and granularly spongy surface of the nanocomposite, thus being highly favorable to the removal of toxic CR dye. The 92.9% was the highest reported adsorption of CR dye on SA-g-p(AM-Bn)/TiO_2_ nanocomposite at optimized conditions (100 mL of 100 mg/L of solution CR dye, SA-g-p(AM-Bn)/TiO_2_ nanocomposite adsorbent dose = 0.05 g, time = 60 min, pH = 7). The best adsorption capacity was 185.92 mg.g^–1^, and CR dye adsorption fitted better with the Freundlich isotherm and the pseudo-second-order model. Moreover, recyclability of SA-g-p(AM-Bn)/TiO_2_ was performed and exhibited 82.2% CR dye adsorption even after six successive adsorption–desorption cycles. Hence, re-cyclable SA-g-p(AM-Bn)/TiO_2_ indicates CR dye adsorption and can be used as an excellent adsorbent in textiles’ dyes.

## Data Availability

The datasets generated and/or analyzed during the current study are available from the corresponding author on reasonable request.
